# Supplementary material for: Disparities in chronic kidney disease burden estimates: From different sources, definitions, and equations
Source: PLoS One. 2025 Aug 25;20(8):e0328653. doi: 10.1371/journal.pone.0328653 (PMC12377590; doi:10.1371/journal.pone.0328653)
Supplement: S1 Fig — (DOCX) [file pone.0328653.s001.docx]

NHANES participants at 1999-2018 (n=101,316)

53,079 excluded

• 46,235 age<20 years old

• 6844 lacking data regarding serum creatinine, urine albumin, or urine creatinine

48,237 participants met the requirements included

• 1999-2000, n=4049

• 2001-2002, n=4619

• 2003-3004, n=4366

•2005-2006, n=4399

•2007-2008, n=5205

•2009-2010, n=5621

•2011-2012, n=4831

•2013-2014, n=5246

•2015-2016, n=5074

•2017-2018, n=4827

S1 Fig. Flow diagram of participants’ inclusion.
