# Supplementary material for: Disparities in chronic kidney disease burden estimates: From different sources, definitions, and equations
Source: PLoS One. 2025 Aug 25;20(8):e0328653. doi: 10.1371/journal.pone.0328653 (PMC12377590; doi:10.1371/journal.pone.0328653)
Supplement: S1 Table — (DOCX) [file pone.0328653.s002.docx]

Table S1. Equations to predict GFR.

| **Name** | **Race** | **Sex** | **Age** | **SCr** | **Equation** |
| --- | --- | --- | --- | --- | --- |
| CKD-EPI_2009_ | Black | Female |  | ≤0.7 | 166 × (Scr/0.7)^-0.329^ × (0.993)^Age^ |
|  |  |  |  | >0.7 | 166 × (Scr/0.7)^-1.209^ × (0.993)^Age^ |
|  |  | Male |  | ≤0.9 | 163 × (Scr/0.9)^-0.411^ × (0.993)^Age^ |
|  |  |  |  | >0.9 | 163 × (Scr/0.9)^-1.209^ × (0.993)^Age^ |
|  | White or other | Female |  | ≤0.7 | 144 × (Scr/0.7)^-0.329^ × (0.993)^Age^ |
|  |  |  |  | >0.7 | 144 × (Scr/0.7)^-1.209^ × (0.993)^Age^ |
|  |  | Male |  | ≤0.9 | 141 × (Scr/0.9)^-0.411^ × (0.993)^Age^ |
|  |  |  |  | >0.9 | 141 × (Scr/0.9)^-1.209^ × (0.993)^Age^ |
| CKD-EPI_2021_ |  | Female |  | ≤0.7 | 143 × (SCr/0.7)^-0.241^ × 0.9938^Age^ |
|  |  |  |  | >0.7 | 143 × (SCr/0.7)^-1.200^ × 0.9938^Age^ |
|  |  | Male |  | ≤0.9 | 142 × (SCr/0.9)^-0.302^ × 0.9938^Age^ |
|  |  |  |  | >0.9 | 142 × (SCr/0.9)^-1.200^ × 0.9938^Age^ |
| EKFC |  |  | 18-40 | <Q_SCr_ | 107.3 × (SCr/Q_SCr_)^-0.322^ |
|  |  |  |  | ≥Q_SCr_ | 107.3 × (SCr/Q_SCr_)^-1.132^ |
|  |  |  | >40 | <Q_SCr_ | 107.3 × (SCr/Q_SCr_)^-0.322^ × 0.990^(Age-40)^ |
|  |  |  |  | ≥Q_SCr_ | 107.3 × (SCr/Q_SCr_)^-1.132^ × 0.990^(Age-40)^ |

Serum creatinine (SCr) expressed as mg/dL while 1 mg/dL equal to 88.4 μmol/L. Q-values (mg/dL) correspond to the median SCr values for the age- and sex-specific populations. In US adults, race-free Q values for males and females are 0.97 mg/dL and 0.73 mg/dL, respectively.
