# Supplementary material for: Disparities in chronic kidney disease burden estimates: From different sources, definitions, and equations
Source: PLoS One. 2025 Aug 25;20(8):e0328653. doi: 10.1371/journal.pone.0328653 (PMC12377590; doi:10.1371/journal.pone.0328653)
Supplement: S2 Table — (DOCX) [file pone.0328653.s003.docx]

S2 Table. Correction for serum creatinine in NHANES.

| **Year** | **Correction for Serum Creatinine** |
| --- | --- |
| 1999-2000 | Standard Creatinine (mg/dL) = 1.013 * NHANES Creatinine (mg/dL) + 0.147 |
| 2001-2002 | No correction is necessary for serum creatinine values |
| 2003-2004 | No correction is necessary for serum creatinine values |
| 2005-2006 | Standard Creatinine (mg/dL) = 0.978 * NHANES Creatinine (mg/dL) - 0.016 |
| 2007 | No correction is necessary for serum creatinine values |
| 2008 | IDMS*-*traceable |
| 2009-2010 | IDMS*-*traceable |
| 2011-2012 | IDMS*-*traceable |
| 2013-2014 | IDMS*-*traceable |
| 2015-2016 | IDMS*-*traceable |
| 2017-2018 | IDMS*-*traceable |
