# Supplementary material for: Disparities in chronic kidney disease burden estimates: From different sources, definitions, and equations
Source: PLoS One. 2025 Aug 25;20(8):e0328653. doi: 10.1371/journal.pone.0328653 (PMC12377590; doi:10.1371/journal.pone.0328653)
Supplement: S3 Table — (DOCX) [file pone.0328653.s004.docx]

S3 Table. Annual estimated number and rate of CKD prevalence in US adults (1999-2018).

| **Sex** | **Year** | **Fixed threshold** | | | | **Age-adapted thresholds** | | |
| --- | --- | --- | --- | --- | --- | --- | --- | --- |
|  |  | GBD^a^ | NHANES^b^ | | | NHANES^b^ | | |
|  |  | CKD-EPI_2009_ | CKD-EPI_2009_ | CKD-EPI_2021_ | EKFC_RF_ | CKD-EPI_2009_ | CKD-EPI_2021_ | EKFC_RF_ |
| Both | 1999 | 25,474,987  12.88 (12.00 to 13.76) | 23,462,321  13.21 (11.02, 15.40) | 22,393,581  12.61 (10.50, 14.72) | 23,929,057  13.47 (11.34, 15.60) | 21,250,255  11.97 (10.03,13.90) | 20,925,700  11.78 (9.91, 13.65) | 21,549,607  12.13 (10.18, 14.09) |
|  | 2000 | 25,878,080  12.94 (12.04 to 13.82) |  |  |  |  |  |  |
|  | 2001 | 26,192,616  12.94 (12.04 to 13.81) | 25,211,644  13.56 (12.05, 15.07) | 23,462,272  12.62 (11.27, 13.97) | 25,581,829  13.76 (12.28, 15.24) | 21,944,459  11.80 (10.50, 13.11) | 21,174,477  11.39 (10.12, 12.66) | 21,355,538  11.49 (10.30, 12.67) |
|  | 2002 | 26,466,164  12.93 (12.03 to 13.78) |  |  |  |  |  |  |
|  | 2003 | 26,743,732  12.91 (12.03 to 13.75) | 26,304,470  13.74 (11.28, 16.20) | 24,658,174  12.88 (10.54, 15.21) | 27,716,393  14.48 (11.77, 17.18) | 21,255,581  11.10 (9.05, 13.15) | 20,569,497  10.74 (8.74, 12.75) | 21,209,848  11.08 (8.95, 13.21) |
|  | 2004 | 27,024,792  12.90 (12.03 to 13.73) |  |  |  |  |  |  |
|  | 2005 | 27,414,107  12.93 (12.06 to 13.75) | 27,613,100  14.11 (11.61, 16.61) | 25,872,624  13.22 (11.09, 15.36) | 28,689,359  14.66 (12.17, 17.16) | 23,805,272  12.17 (10.20, 14.13) | 22,574,486  11.54 (9.83, 13.24) | 23,648,538  12.09 (10.27, 13.90) |
|  | 2006 | 27,969,546  13.03 (12.16 to 13.87) |  |  |  |  |  |  |
|  | 2007 | 28,762,150  13.24 (12.35 to 14.11) | 27,783,001  13.98 (12.50, 15.46) | 25,790,418  12.98 (11.83, 14.13) | 28,256,877  14.22 (13.01, 15.44) | 24,281,718  12.22 (10.82, 13.63) | 23,170,740  11.66 (10.38, 12.95) | 24,067,086  12.11 (11.02, 13.21) |
|  | 2008 | 29,674,986  13.50 (12.58 to 14.38) |  |  |  |  |  |  |
|  | 2009 | 30,528,216  13.72 (12.78 to 14.63) | 26,077,395  12.72 (11.10, 14.33) | 24,223,569  11.81 (10.32, 13.31) | 26,753,690  13.05 (11.42, 14.67) | 21,770,159  10.62 (9.20, 12.03) | 20,970,297  10.23 (8.81, 11.64) | 22,089,594  10.77 (9.32, 12.22) |
|  | 2010 | 31,276,799  13.87 (12.93 to 14.80) |  |  |  |  |  |  |
|  | 2011 | 31,878,822  13.96 (13.01 to 14.91) | 29,325,231  14.06 (11.72, 16.40) | 27,938,101  13.40 (11.09, 15.70) | 30,559,934  14.65 (12.41, 16.89) | 25,725,845  12.33 (10.38, 14.29) | 25,245,058  12.10 (10.20, 14.01) | 26,323,528  12.62 (10.90, 14.34) |
|  | 2012 | 32,527,244  14.08 (13.10 to 15.02) |  |  |  |  |  |  |
|  | 2013 | 33,231,272  14.22 (13.21 to 15.16) | 33,578,167  15.45 (13.72, 17.18) | 31,520,240  14.50 (12.93, 16.07) | 33,814,375  15.56 (13.84, 17.27) | 28,759,809  13.23 (11.63, 14.83) | 27,653,678  12.72 (11.30, 14.14) | 28,159,614  12.96 (11.40, 14.51) |
|  | 2014 | 33,926,554  14.36 (13.33 to 15.32) |  |  |  |  |  |  |
|  | 2015 | 34,697,138  14.53 (13.49 to 15.50) | 31,284,622  14.22 (12.01, 16.43) | 29,355,026  13.34 (11.43, 15.25) | 32,415,504  14.73 (12.67, 16.79) | 27,464,730  12.48 (10.55, 14.41) | 26,874,210  12.21 (10.34, 14.09) | 27,524,232  12.51 (10.74, 14.28) |
|  | 2016 | 35,486,924  14.72 (13.64 to 15.70) |  |  |  |  |  |  |
|  | 2017 | 36,373,783  14.95 (13.84 to 15.95) | 33,301,207  14.94 (12.97, 16.91) | 31,337,901  14.06 (12.41, 15.71) | 34,698,586  15.57 (13.44, 17.69) | 28,144,513  12.63 (10.95, 14.30) | 26,829,713  12.04 (10.48, 13.59) | 28,093,657  12.60 (10.96, 14.24) |
|  | 2018 | 37,261,441  15.17 (14.04 to 16.19) |  |  |  |  |  |  |
| Males | 1999 | 10,130,387  10.63 (9.87 to 11.36) | 9,442,355  11.07 (9.46, 12.68) | 9,087,972  10.66 (9.12, 12.20) | 9,358,883  10.97 (9.38, 12.57) | 8,804,458  10.32 (9.03, 11.62) | 8,736,048  10.24 (8.91, 11.58) | 8,900,982  10.44 (9.12, 11.75) |
|  | 2000 | 10,331,521  10.70 (9.94 to 11.43) |  |  |  |  |  |  |
|  | 2001 | 10,487,806  10.73 (9.98 to 11.44) | 11,006,531  12.29 (10.95, 13.64) | 10,058,843  11.23 (9.90, 12.57) | 10,684,099  11.93 (10.48, 13.38) | 9,628,442  10.75 (9.23, 12.27) | 9,239,685  10.32 (8.87, 11.76) | 8,881,093  9.92 (8.47, 11.37) |
|  | 2002 | 10,621,026  10.73 (9.99 to 11.43) |  |  |  |  |  |  |
|  | 2003 | 10,753,374  10.73 (9.99 to 11.43) | 11,787,651  12.74 (11.26, 14.21) | 11,071,620  11.96 (10.48, 13.45) | 11,855,748  12.81 (11.34, 14.28) | 9,970,726  10.77 (9.20, 12.35) | 9,892,589  10.69 (9.09, 12.28) | 9,636,700  10.41 (9.02, 11.81) |
|  | 2004 | 10,886,101  10.73 (9.99 to 11.42) |  |  |  |  |  |  |
|  | 2005 | 11,068,580  10.78 (10.03 to 11.47) | 11,528,714  12.23 (9.66, 14.81) | 10,437,106  11.07 (8.78, 13.37) | 11,067,567  11.74 (9.24, 14.25) | 10,155,314  10.78 (8.44, 13.11) | 9,372,448  9.95 (7.65, 12.24) | 9,408,954  9.98 (7.74, 12.23) |
|  | 2006 | 11,311,087  10.88 (10.12 to 11.58) |  |  |  |  |  |  |
|  | 2007 | 11,639,977  11.06 (10.28 to 11.78) | 11,275,548  11.76 (10.58, 12.93) | 10,597,809  11.05 (10.09, 12.01) | 10,661,054  11.12 (10.16, 12.07) | 10,455,579  10.90 (9.67, 12.13) | 9,941,527  10.37 (9.39, 11.34) | 9,572,211  9.98 (9.15, 10.82) |
|  | 2008 | 12,017,435  11.27 (10.47 to 12.03) |  |  |  |  |  |  |
|  | 2009 | 12,376,097  11.46 (10.64 to 12.25) | 11,544,695  11.63 (10.53, 12.74) | 10,584,597  10.66 (9.62, 11.70) | 11,274,769  11.36 (10.26, 12.45) | 9,836,400  9.91 (8.83, 10.98) | 9,431,665  9.50 (8.54, 10.46) | 9,396,809  9.47 (8.49, 10.45) |
|  | 2010 | 12,710,323  11.62 (10.78 to 12.41) |  |  |  |  |  |  |
|  | 2011 | 12,998,554  11.73 (10.89 to 12.53) | 13,002,750  12.87 (10.43, 15.32) | 12,495,988  12.37 (9.86, 14.89) | 13,197,959  13.07 (10.51, 15.63) | 11,187,409  11.08 (8.99, 13.16) | 11,107,182  11.00 (8.98, 13.01) | 11,232,064  11.12 (9.15, 13.09) |
|  | 2012 | 13,316,184  11.87 (11.02 to 12.67) |  |  |  |  |  |  |
|  | 2013 | 13,658,052  12.03 (11.16 to 12.82) | 13,886,080  13.22 (11.71, 14.73) | 12,737,037  12.12 (10.65, 13.60) | 13,152,549  12.52 (11.02, 14.02) | 11,542,209  10.99 (9.50, 12.47) | 11,061,043  10.53 (9.07, 11.99) | 10,706,572  10.19 (8.80, 11.59) |
|  | 2014 | 13,990,096  12.19 (11.31 to 12.98) |  |  |  |  |  |  |
|  | 2015 | 14,347,740  12.37 (11.47 to 13.18) | 13,331,125  12.58 (10.30, 14.86) | 12,520,767  11.82 (9.47, 14.16) | 13,070,241  12.33 (10.08, 14.59) | 12,136,965  11.45 (9.27, 13.63) | 11,786,192  11.12 (8.75, 13.49) | 11,671,383  11.01 (8.82, 13.21) |
|  | 2016 | 14,686,261  12.54 (11.63 to 13.35) |  |  |  |  |  |  |
|  | 2017 | 15,038,910  12.73 (11.78 to 13.55) | 15,115,785  14.06 (12.20, 15.91) | 13,981,739  13.00 (11.29, 14.71) | 14,762,027  13.73 (12.00, 15.46) | 13,193,436  12.27 (10.49, 14.05) | 12,733,911  11.84 (10.01, 13.68) | 12,586,416  11.71 (9.86, 13.55) |
|  | 2018 | 15,379,980  12.90 (11.92 to 13.77) |  |  |  |  |  |  |
| Females | 1999 | 15,344,600  14.99 (13.98 to 15.99) | 14,019,966  15.19 (13.53, 16.84) | 13,305,609  14.41 (12.76, 16.07) | 14,570,174  15.78 (14.22, 17.35) | 12,445,796  13.48 (11.94, 15.02) | 12,189,652  13.20 (11.68, 14.73) | 12,648,625  13.70 (12.14, 15.26) |
|  | 2000 | 15,546,559  15.02 (14.01 to 16.04) |  |  |  |  |  |  |
|  | 2001 | 15,704,810  15.01 (14.01 to 16.01) | 14,205,112  14.74 (13.34, 16.15) | 13,403,429  13.91 (12.65, 15.17) | 14,897,730  15.46 (14.16, 16.76) | 12,316,017  12.78 (11.50, 14.07) | 11,934,792  12.39 (11.01, 13.76) | 12,474,445  12.95 (11.78, 14.11) |
|  | 2002 | 15,845,138  14.98 (13.99 to 15.97) |  |  |  |  |  |  |
|  | 2003 | 15,990,358  14.96 (13.96 to 15.93) | 14,516,820  14.67 (13.14, 16.21) | 13,586,553  13.73 (12.01, 15.46) | 15,860,644  16.03 (13.90, 18.17) | 11,284,854  11.41 (10.14, 12.67) | 10,676,908  10.79 (9.48, 12.10) | 11,573,148  11.70 (10.04, 13.36) |
|  | 2004 | 16,138,691  14.93 (13.93 to 15.89) |  |  |  |  |  |  |
|  | 2005 | 16,345,527  14.95 (13.94 to 15.91) | 16,084,385  15.86 (13.45, 18.26) | 15,435,518  15.22 (12.90, 17.54) | 17,621,793  17.37 (14.94, 19.81) | 13,649,957  13.46 (11.77, 15.15) | 13,202,038  13.02 (11.53, 14.51) | 14,239,584  14.04 (12.35, 15.73) |
|  | 2006 | 16,658,459  15.06 (14.05 to 16.01) |  |  |  |  |  |  |
|  | 2007 | 17,122,173  15.30 (14.29 to 16.27) | 16,507,453  16.06 (14.16, 17.97) | 15,192,609  14.78 (12.92, 16.65) | 17,595,823  17.12 (14.97, 19.27) | 13,826,139  13.45 (11.64, 15.26) | 13,229,213  12.87 (10.84, 14.90) | 14,494,874  14.10 (12.18, 16.02) |
|  | 2008 | 17,657,551  15.60 (14.59 to 16.59) |  |  |  |  |  |  |
|  | 2009 | 18,152,118  15.84 (14.84 to 16.87) | 14,532,700  13.73 (12.22, 15.25) | 13,638,973  12.89 (11.33, 14.45) | 15,478,922  14.63 (13.18, 16.08) | 11,933,759  11.28 (9.56, 12.99) | 11,538,632  10.91 (9.18, 12.63) | 12,692,784  12.00 (10.29, 13.70) |
|  | 2010 | 18,566,476  16.00 (15.00 to 17.03) |  |  |  |  |  |  |
|  | 2011 | 18,880,268  16.07 (15.04 to 17.11) | 16,322,482  15.18 (13.53, 16.83) | 15,442,113  14.36 (12.70, 16.02) | 17,361,975  16.14 (14.24, 18.05) | 14,538,436  13.52 (11.71, 15.33) | 14,137,876  13.14 (11.41, 14.88) | 15,091,464  14.03 (12.44, 15.62) |
|  | 2012 | 19,211,060  16.16 (15.09 to 17.21) |  |  |  |  |  |  |
|  | 2013 | 19,573,221  16.28 (15.18 to 17.34) | 19,692,086  17.53 (14.73, 20.33) | 18,783,203  16.73 (14.22, 19.23) | 20,661,826  18.40 (15.62, 21.18) | 17,217,600  15.33 (12.65, 18.01) | 16,592,635  14.77 (12.46, 17.09) | 17,453,042  15.54 (13.00, 18.08) |
|  | 2014 | 19,936,458  16.40 (15.28 to 17.49) |  |  |  |  |  |  |
|  | 2015 | 20,349,398  16.57 (15.42 to 17.69) | 17,953,497  15.74 (13.13, 18.35) | 16,834,260  14.76 (12.55, 16.97) | 19,345,264  16.96 (14.59, 19.32) | 15,327,765  13.44 (11.32, 15.56) | 15,088,018  13.23 (11.23, 15.23) | 15,852,849  13.90 (11.86, 15.93) |
|  | 2016 | 20,800,663  16.77 (15.60 to 17.92) |  |  |  |  |  |  |
|  | 2017 | 21,334,874  17.04 (15.85 to 18.21) | 18,185,422  15.76 (13.49, 18.02) | 17,356,162  15.04 (13.08, 17.00) | 19,936,559  17.27 (14.79, 19.76) | 14,951,077  12.95 (10.81, 15.10) | 14,095,803  12.21 (10.48, 13.95) | 15,507,241  13.44 (11.33, 15.55) |
|  | 2018 | 21,881,462  17.32 (16.10 to 18.49) |  |  |  |  |  |  |

^a^ The estimated prevalence rates (per 100,000 population) are presented using number and 95% uncertainty intervals (UIs) in the GBD study.

^b^ The estimated prevalence rates (per 100 population) are presented using number and 95% confidence interval (CIs) in the NHANES.
