# Supplementary material for: Disparities in chronic kidney disease burden estimates: From different sources, definitions, and equations
Source: PLoS One. 2025 Aug 25;20(8):e0328653. doi: 10.1371/journal.pone.0328653 (PMC12377590; doi:10.1371/journal.pone.0328653)
Supplement: S4 Table — (DOCX) [file pone.0328653.s005.docx]

S4 Table. Reported results and corresponding GBD estimates of CKD prevalence across different countries.

| **Area** | **Year** | **GBD estimate (95%UI)** | **Reported data (95%CI)** | **Source** |
| --- | --- | --- | --- | --- |
| Australia | 1999 | ≥25 years: 11.2 (10.6, 11.9)  Males: 8.5 (8.0, 9.1)  Females: 13.8 (13.0, 14.6) | ≥25 years: 11.5 (9.4, 14.1)  Males: 10.9 (9.0, 13.1)  Females: 12.2 (9.6, 15.3) | Australian Diabetes, Obesity and Lifestyle (AusDiab) Study [1] |
|  | 2000 | ≥25 years: 11.3 (10.7, 11.0)  Males: 8.6 (8.0, 9.2)  Females: 13.8 (13.1, 14.7) |  |  |
| Australia | 2020 | ≥20 years: 11.7 (10.9, 12.5)  Males: 9.5 (8.8, 10.2)  Females: 13.8 (12.8, 14.8) | ≥18 years: 8.4  Males: 9.8  Females: 7.5 | MedicineInsight [2] |
| Canada | 2007 | ≥20 years: 11.6 (11.1, 12.2)  Males: 10.9 (10.4, 11.4)  Females: 12.3 (11.8, 13.0) | 18-79 years: 12.5  Males: 12.4  Females: 12.7 | Canadian Health Measures Survey [3] |
|  | 2008 | ≥20 years: 11.7 (11.2, 12.2)  Males: 10.9 (10.4, 11.4)  Females: 12.4 (11.9, 13.0) |  |  |
|  | 2009 | ≥20 years: 11.8 (11.2, 12.3)  Males: 11.0 (10.5, 11.5)  Females: 12.5 (11.9, 13.1) |  |  |
| China | 2018 | ≥20 years: 10.3 (9.6, 11.1)  Males: 9.3 (8.6, 10.0)  Females: 11.4 (10.6, 12.3) | ≥18 years: 8.2 (7.8, 8.6)  Males: 7.7 (7.2, 8.1)  Females: 8.8 (8.3, 9.3) | Sixth China Chronic Disease and Risk Factor Surveillance (CCDRFS) [4] |
|  | 2019 | ≥20 years: 10.4 (9.6, 11.2)  Males: 9.3 (8.6, 10.1)  Females: 11.5 (10.6, 12.4) |  |  |
| Iceland | 2008 | ≥20 years: 7.9 (7.4, 8.5)  Males: 6.9 (6.5, 7.3)  Females: 9.0 (8.3, 9.6) | ≥18 years: 11.6 (11.5, 11.7)  Males: 10.6 (10.6, 10.7)  Females: 12.6 (12.5, 12.7) | All healthcare institutions in Iceland in 2008-2016 [5] |
|  | 2009 | ≥20 years: 7.9 (7.4, 8.5)  Males: 6.9 (6.5, 7.4)  Females: 9.0 (8.3, 9.7) |  |  |
|  | 2010 | ≥20 years: 8.0 (7.5, 8.6)  Males: 7.0 (6.6, 7.5)  Females: 9.0 (8.4, 9.7) |  |  |
|  | 2011 | ≥20 years: 8.1 (7.5, 8.6)  Males: 7.1 (6.7, 7.6)  Females: 9.0 (8.4, 9.6) |  |  |
|  | 2012 | ≥20 years: 8.1 (7.6, 8.6)  Males: 7.2 (6.8, 7.7)  Females: 8.9 (8.3, 9.5) |  |  |
|  | 2013 | ≥20 years: 8.0 (7.6, 8.5)  Males: 7.3 (6.9, 7.7)  Females: 8.8 (8.2, 9.3) |  |  |
|  | 2014 | ≥20 years: 8.0 (7.6, 8.5)  Males: 7.4 (7.0, 7.8)  Females: 8.6 (8.1, 9.2) |  |  |
|  | 2015 | ≥20 years: 8.0 (7.6, 8.5)  Males: 7.4 (7.0, 7.9)  Females: 8.6 (8.1, 9.1) |  |  |
|  | 2016 | ≥20 years: 8.1 (7.6, 8.6)  Males: 7.4 (7.0, 7.9)  Females: 8.8 (8.2, 9.3) |  |  |
| Korea | 2007 | ≥20 years: 10.8 (10.2, 11.5)  Males: 9.3 (8.8, 9.9)  Females: 12.1 (11.5, 12.9) | ≥19 years: 5.1 (4.7, 5.5)  Males: 4.8 (4.3, 5.3)  Females: 5.4 (4.9, 5.9) | Korea National Health and Nutrition Examination Survey (KNHANES) [6] |
|  | 2008 | ≥20 years: 10.8 (10.2, 11.4)  Males: 9.4 (8.8, 9.9)  Females: 12.1 (11.5, 12.8) |  |  |
|  | 2009 | ≥20 years: 10.8 (10.2, 11.3)  Males: 9.4 (8.9, 9.9)  Females: 12.1 (11.5, 12.8) |  |  |
|  | 2010 | ≥20 years: 10.8 (10.3, 11.4)  Males: 9.4 (8.9, 9.9)  Females: 12.1 (11.5, 12.9) |  |  |
|  | 2011 | ≥20 years: 10.9 (10.3, 11.5)  Males: 9.5 (9.0, 10.0)  Females: 12.3 (11.6, 13.1) | ≥19 years: 5.4 (5.0, 5.9)  Males: 4.2 (3.7, 4.6)  Females: 5.8 (5.1, 6.4) |  |
|  | 2012 | ≥20 years: 11.0 (10.4, 11.6)  Males: 9.5 (9.1, 10.1)  Females: 12.4 (11.8, 13.3) |  |  |
|  | 2013 | ≥20 years: 11.1 (10.6, 11.8)  Males: 9.6 (9.1, 10.1)  Females: 12.6 (11.9, 13.4) |  |  |
|  | 2014 | ≥20 years: 11.2 (10.7, 11.9)  Males: 9.7 (9.2, 10.2)  Females: 12.7 (12.0, 13.6) | ≥19 years: 7.1 (6.6, 7.6)  Males: 6.3 (5.7, 6.9)  Females: 7.9 (7.2, 8.6) |  |
|  | 2015 | ≥20 years: 11.4 (10.8, 12.1)  Males: 9.8 (9.3, 10.4)  Females: 12.9 (12.1, 13.8) |  |  |
|  | 2016 | ≥20 years: 11.5 (10.9, 12.2)  Males: 10.0 (9.5, 10.5)  Females: 13.0 (12.3, 14.0) |  |  |
|  | 2017 | ≥20 years: 11.7 (11.1, 12.4)  Males: 10.2 (9.7, 10.8)  Females: 13.2 (12.4, 14.1) | ≥19 years: 7.1 (6.6, 7.6)  Males: 7.4 (6.8, 8.1)  Females: 6.8 (6.2, 7.4) |  |
|  | 2018 | ≥20 years: 11.8 (11.2, 12.6)  Males: 10.4 (9.9, 11.0)  Females: 13.3 (12.5, 14.2) |  |  |
|  | 2019 | ≥20 years: 12.0 (11.4, 12.8)  Males: 10.6 (10.0, 11.2)  Females: 13.5 (12.7, 14.4) |  |  |
| Luxembourg | 2007 | ≥20 years: 10.6 (9.9, 11.4) | 18-69 years: 5.9 | Observation of Cardiovascular Risk Factors in Luxembourg (ORISCAV-LUX) [7] |
|  | 2008 | ≥20 years: 10.6 (9.9, 11.5) |  |  |
| Malaysia | 2017 | ≥20 years: 15.4 (14.2, 16.8) | ≥18 years: 15.5 (12.3, 19.3) | A nationwide population-based cross-sectional study [8] |
|  | 2018 | ≥20 years: 15.4 (14.3, 16.8) |  |  |
| Netherlands | 1996 | ≥20 years: 10.8 (10.0, 11.8) | ≥20 years: 6.7 (6.6, 6.7) | Integrated Primary Care Information (IPCI) project [9] |
|  | 1997 | ≥20 years: 10.9 (10.1, 12.0) |  |  |
|  | 1998 | ≥20 years: 11.0 (10.2, 12.1) |  |  |
|  | 1999 | ≥20 years: 11.1 (10.3, 12.3) |  |  |
|  | 2000 | ≥20 years: 11.1 (10.3, 12.4) |  |  |
|  | 2001 | ≥20 years: 11.1 (10.4, 12.3) |  |  |
|  | 2002 | ≥20 years: 11.1 (10.4, 12.0) |  |  |
|  | 2003 | ≥20 years: 11.0 (10.3, 11.9) |  |  |
|  | 2004 | ≥20 years: 11.0 (10.3, 11.8) |  |  |
|  | 2005 | ≥20 years: 11.0 (10.3, 11.8) |  |  |
|  | 2006 | ≥20 years: 11.1 (10.4, 11.8) |  |  |
|  | 2007 | ≥20 years: 11.2 (10.5, 11.9) |  |  |
|  | 2008 | ≥20 years: 11.3 (10.6, 12.0) |  |  |
|  | 2009 | ≥20 years: 11.4 (10.6, 12.1) |  |  |
|  | 2010 | ≥20 years: 11.5 (10.7, 12.2) |  |  |
|  | 2011 | ≥20 years: 11.6 (10.8, 12.3) |  |  |
| Netherlands | 2019 | ≥20 years: 12.5 (11.7, 13.4) | ≥18 years: 8.9 | PHARMO Data Network [10] |
| Poland | 2011 | ≥20 years: 11.4 (10.6, 12.2)  Males: 9.0 (8.4, 9.7)  Females: 13.5 (10.6, 12.2) | 18-79 years: 5.8 (4.6, 7.2)  Males: 5.3 (4.1, 6.8)  Females: 6.2 (4.4, 8.7) | NATPOL 2011 survey [11] |
| Portugal | 2017 | ≥20 years: 10.4 (9.4, 11.1)  Males: 9.2 (8.5, 9.9)  Females: 11.4 (10.2, 12.2) | ≥18 years: 20.9 (6.5, 35.3)  Males: 20.0 (0.0, 31.1)  Females: 21.7 (12.3, 31.0) | RENA study [12] |
|  | 2018 | ≥20 years: 10.5 (9.5, 11.3)  Males: 9.3 (8.5, 10.0)  Females: 11.5 (10.3, 12.4) |  |  |
| Romania | 2012 | ≥20 years: 12.0 (11.3, 12.6)  Males: 10.2 (9.7, 10.7)  Females: 13.5 (12.8, 14.3) | 20-79 years: 6.7 (5.6, 7.9)  Males: 6.7 (5.6, 7.9)  Females: 6.8 (5.6, 7.9) | PREDATORR study [13] |
|  | 2013 | ≥20 years: 12.1 (11.4, 12.7)  Males: 10.3 (9.8, 10.9)  Females: 13.7 (12.9, 14.4) |  |  |
|  | 2014 | ≥20 years: 12.2 (11.6, 12.8)  Males: 10.5 (10.0, 11.1)  Females: 13.8 (13.0, 14.6) |  |  |
| Spain | 2008 | ≥20 years: 10.0 (9.5, 10.6)  Males: 8.4 (8.0, 8.9)  Females: 11.6 (10.8, 12.3) | ≥18 years: 15.1 (14.3, 16.0)  Males: 23.1 (22.0, 24.2)  Females: 7.3 (6.6, 8.0) | A study on nutrition and cardiovascular risk in Spain (ENRICA) [14] |
|  | 2009 | ≥20 years: 10.1 (9.5, 10.7)  Males: 8.5 (7.9, 9.0)  Females: 11.7 (10.9, 12.5) |  |  |
|  | 2010 | ≥20 years: 10.2 (9.5, 10.8)  Males: 8.5 (7.9, 9.2)  Females: 11.8 (10.9, 12.6) |  |  |
| Switzerland | 2010 | ≥20 years: 13.2 (12.4, 14.1)  Males: 10.2 (9.6, 10.9)  Females: 16.1 (15.0, 17.2) | ≥15 years: 10.4  Males: 10.3  Females: 10.5 | Swiss Survey on Salt (SSS) [15] |
|  | 2011 | ≥20 years: 13.2 (12.4, 14.0)  Males: 10.2 (9.6, 10.9)  Females: 16.1 (15.0, 17.1) |  |  |
|  | 2012 | ≥20 years: 13.2 (12.4, 13.9)  Males: 10.2 (9.6, 10.8)  Females: 16.1 (15.0, 17.0) |  |  |
| Switzerland | 2010 | ≥20 years: 13.2 (12.4, 14.1) | ≥15 years: about 18 | A Swiss cross-sectional study in the primary care setting [16] |
| United Kingdom | 2009 | ≥20 years: 11.4 (10.6, 12.1) | ≥16 years: 12.6 (11.9, 13.4) | Health Surveys for England (HSE) [17] |
|  | 2010 | ≥20 years: 11.4 (10.6, 12.1) |  |  |
|  | 2016 | ≥20 years: 11.1 (10.4, 11.8) | ≥16 years: 13.9 (12.8, 15.2) |  |

References

1. White SL, Polkinghorne KR, Atkins RC, Chadban SJ. Comparison of the prevalence and mortality risk of CKD in Australia using the CKD Epidemiology Collaboration (CKD-EPI) and Modification of Diet in Renal Disease (MDRD) Study GFR estimating equations: the AusDiab (Australian Diabetes, Obesity and Lifestyle) Study. Am J Kidney Dis 2010;55(4):660-70.

2. Jun M, Wick J, Neuen BL, et al. The Prevalence of CKD in Australian Primary Care: Analysis of a National General Practice Dataset. Kidney Int Rep 2023;9(2):312-322.

3. Arora P, Vasa P, Brenner D, et al. Prevalence estimates of chronic kidney disease in Canada: results of a nationally representative survey. CMAJ 2013;185(9):E417-23.

4. Wang L, Xu X, Zhang M, et al. Prevalence of Chronic Kidney Disease in China: Results From the Sixth China Chronic Disease and Risk Factor Surveillance. JAMA Intern Med 2023;183(4):298-310.

5. Jonsson AJ, Lund SH, Eriksen BO, Palsson R, Indridason OS. The prevalence of chronic kidney disease in Iceland according to KDIGO criteria and age-adapted estimated glomerular filtration rate thresholds. Kidney Int 2020;98(5):1286-1295.

6. Yoon SY, Park HW, Kim HJ, et al. National trends in the prevalence of chronic kidney disease among Korean adults, 2007-2020. Sci Rep 2023;13(1):5831.

7. Alkerwi A, Sauvageot N, El Bahi I et al. Prevalence and related risk factors of chronic kidney disease among adults in Luxembourg: evidence from the observation of cardiovascular risk factors (ORISCAV-LUX) study. BMC Nephrol 2017;18(1):358.

8. Saminathan TA, Hooi LS, Mohd Yusoff MF, et al. Prevalence of chronic kidney disease and its associated factors in Malaysia; findings from a nationwide population-based cross-sectional study. BMC Nephrol 2020;21(1):344.

9. van Blijderveen JC, Straus SM, Zietse R, Stricker BH, Sturkenboom MC, Verhamme KM. A population-based study on the prevalence and incidence of chronic kidney disease in the Netherlands. Int Urol Nephrol 2014;46(3):583-92.

10. Vervloet MG, de Jong HJ, Pander J, Overbeek JA. Prevalence of chronic kidney disease in the Netherlands and its cardiovascular and renal complications. BMC Nephrol 2023;24(1):337.

11. Zdrojewski Ł, Zdrojewski T, Rutkowski M, et al. Prevalence of chronic kidney disease in a representative sample of the Polish population: results of the NATPOL 2011 survey. Nephrol Dial Transplant 2016;31(3):433-9.

12. Vinhas J, Aires I, Batista C, et al. RENA Study: Cross-Sectional Study to Evaluate CKD Prevalence in Portugal. Nephron 2020;144(10):479-487.

13. Moţa E, Popa SG, Moţa M, et al. Prevalence of chronic kidney disease and its association with cardio-metabolic risk factors in the adult Romanian population: the PREDATORR study. Int Urol Nephrol 2015;47(11):1831-8.

14. Gorostidi M, Sánchez-Martínez M, Ruilope LM, et al. Chronic kidney disease in Spain: Prevalence and impact of accumulation of cardiovascular risk factors. Nefrologia (Engl Ed) 2018;38(6):606-615.

15. Forni Ogna V, Ogna A, Ponte B, et al. Prevalence and determinants of chronic kidney disease in the Swiss population. Swiss Med Wkly 2016;146:w14313.

16. Tomonaga Y, Risch L, Szucs TD, Ambühl PM. The prevalence of chronic kidney disease in a primary care setting: a Swiss cross-sectional study. PLoS One 2013;8(7):e67848.

17. Hounkpatin HO, Harris S, Fraser SDS, et al. Prevalence of chronic kidney disease in adults in England: comparison of nationally representative cross-sectional surveys from 2003 to 2016. BMJ Open 2020;10(8):e038423.
