# Supplementary material for: Disparities in chronic kidney disease burden estimates: From different sources, definitions, and equations
Source: PLoS One. 2025 Aug 25;20(8):e0328653. doi: 10.1371/journal.pone.0328653 (PMC12377590; doi:10.1371/journal.pone.0328653)
Supplement: S1 File — (DOCX) [file pone.0328653.s006.docx]

**Literature Review**

We searched PubMed between the year 2009 to March 13, 2025, for studies evaluating CKD prevalence. Our search strategy utilized the following terms: ((prevalence [Title]) AND ((chronic kidney disease [Title]) OR (CKD [Title]))).

In total, 1037 articles were retrieved. Titles and abstracts of each article were reviewed to determine potentially eligible studies, resulting in the exclusion of 576 articles. Then we read the full text and excluded studies based on the following criteria: (1) the study population was not representative of the general population, (2) the study sample lacked national representativeness, (3) the study did not include adults of all ages, (4) CKD was not defined based on GFR and ACR levels, (5) eGFR was not calculated using the CKD-EPI equation.

The detailed process of identifying and selecting studies is shown in S2 Fig. A total of 26 studies from 16 countries met all inclusion criteria and reported estimated CKD prevalence. The extracted data included geographic region, year, CKD prevalence for the overall population and sex subgroups, and data sources. We compared them with the corresponding GBD estimates (Table S4).

Records from PubMed (n=1037)

Unrelated records excluded (n=576)

Titles and abstracts reviewed (n=1037)

Full-texts articles reviewed (n=461)

435 articles excluded due to:

• Study population was not representative for the general population (n=220)

• Sample lacked national representativeness (n=156)

• Study did not include adults of all ages (n=16)

• CKD was not defined based on GFR and ACR levels (n=28)

• eGFR was not calculated using the CKD-EPI equation (n=15)

Studies included (n=26)

**Identification**

**Screening**

**Included**

**Eligibility**

S2 Fig. Flow diagram of study identification, inclusion, and exclusion.
